# Associations between active commuting and physical and mental wellbeing^☆^

**DOI:** 10.1016/j.ypmed.2013.04.008

**Published:** 2013-08

**Authors:** David K. Humphreys, Anna Goodman, David Ogilvie

**Affiliations:** a UKCRC Centre for Diet and Activity Research (CEDAR), Institute of Public Health, University of Cambridge, UK; b Faculty of Epidemiology and Population Health, London School of Hygiene and Tropical Medicine, London, UK; c MRC Epidemiology Unit, Cambridge, UK

**Keywords:** Active commuting, Physical activity, Wellbeing

## Abstract

**Objective:**

To examine whether a relationship exists between active commuting and physical and mental wellbeing.

**Method:**

In 2009, cross-sectional postal questionnaire data were collected from a sample of working adults (aged 16 and over) in the Commuting and Health in Cambridge study. Travel behaviour and physical activity were ascertained using the Recent Physical Activity Questionnaire (RPAQ) and a seven-day travel-to-work recall instrument from which weekly time spent in active commuting (walking and cycling) was derived. Physical and mental wellbeing were assessed using the Medical Outcomes Study Short Form survey (SF-8). Associations were tested using multivariable linear regression.

**Results:**

An association was observed between physical wellbeing (PCS-8) score and time spent in active commuting after adjustment for other physical activity (adjusted regression coefficients 0.48, 0.79 and 1.21 for 30–149 min/week, 150–224 min/week and ≥ 225 min/week respectively versus < 30 min/week, p = 0.01 for trend; n = 989). No such relationship was found for mental wellbeing (MCS-8) (p = 0.52).

**Conclusion:**

Greater time spent actively commuting is associated with higher levels of physical wellbeing. Longitudinal studies should examine the contribution of changing levels of active commuting and other forms of physical activity to overall health and wellbeing.

## Highlights

• We assessed the relationship between active commuting and physical and mental wellbeing.
• Greater active commuting was associated with higher levels of physical wellbeing.
• The strongest association occurred with at least 45 min of active commuting per day.
• There was no significant association between active commuting and mental wellbeing.

## Introduction

Regular physical activity can contribute to a broad range of health benefits (Biddle and Mutrie, 2008). Consistent associations have been found between physical activity and different aspects of physical and mental wellbeing, including depression and anxiety (Dunn et al., 2005), self-reported wellbeing (Anoyke et al., 2012; Bize et al., 2007; Hamer and Stamatakis, 2010), and emotion and mood (Stathopoulou et al., 2006). Some studies suggest a dose–response relationship (Dunn et al., 2005; Hamer et al., 2009).

This evidence is primarily drawn from studies examining associations with recreational physical activity, rather than more routine activities such as walking and cycling to work (‘active commuting’) (Mutrie and Faulkner, 2004). Qualitative research suggests that choice of travel mode may affect wellbeing (Guell and Ogilvie, 2013; Hiscock et al., 2002) and the nature and intensity of active commuting (AC) may differ from that of recreational physical activity. For example, AC is often solitary and may be experienced as less enjoyable and more stressful than leisure activities. This study uses a validated self-report measure of health-related quality of life (SF-8) to explore the relationship between AC and physical and mental wellbeing in a sample of working adults.

## Methods

### Study setting and data collection

This analysis uses cross-sectional data from the Commuting and Health in Cambridge study, which has previously been described in detail in Ogilvie et al. (2010). The study was set in the city of Cambridge, UK (approximate population: 108,000) and the surrounding area. Commuters aged 16 and over were recruited from multiple workplaces in the city. Between May and October 2009, participants completed postal questionnaires covering their travel behaviour, physical activity and wellbeing. The Hertfordshire Research Ethics Committee granted ethical approval and participants provided written informed consent.

### Outcome measures

Physical and mental wellbeing summary variables were derived from responses to the Medical Outcomes Study Short Form (SF-8). This comprises eight ordinal response questions asking about participants' physical and mental health in the last 4 weeks (general health, physical functioning, role physical, bodily pain, vitality, social functioning, role emotional, and mental health). These were used to create physical (PCS) and mental (MCS) summary scores, which were then scaled to population norms using the methods described in Ware et al. (2001).

### Exposure measure

Time spent actively commuting was derived using an instrument to record participants' self-reported travel to and from work over the previous seven days (Panter et al., 2011) based on a measure shown to have acceptable test-retest reliability (Shannon et al., 2006). Although the exposure was assessed over a different time period (seven days) than that for the outcome (four weeks), the typical weekly cyclical pattern of AC probably makes a seven-day measure more accurate and less susceptible to recall bias. The distribution of AC was heavily skewed: many participants reported little or no time spent actively commuting. We categorised AC a priori into a 4-level categorical variable; 0–29 min/week (‘very low’); 30–149 min/week (‘some AC, but below physical activity guidelines’ (Cheif Medical Officers, 2011)); 150–224 min/week (‘AC above guidelines’); and ≥ 225 min/week (‘very high’) (Yang et al., 2012).

### Covariates

We adjusted our analysis for covariates known to be related to the prevalence of AC (Trost et al., 2002). Participants provided information on their gender, age (grouped as 16–29, 30–39, 40–49, 50–59, ≥ 60 years) and highest educational attainment (dichotomised into ‘less than bachelor's degree’ and ‘bachelor's degree or higher’) and the distance between their home and workplace (kilometres). We calculated body mass index from self-reported weight and height (kg/m^2^) and used standard cutpoints to categorise it into ‘normal or underweight’, ‘overweight’, and ‘obese’ (World Health Organisation, 2000). To control for time spent in other forms of physical activity, we used responses to the validated Recent Physical Activity Questionnaire (RPAQ) (Besson et al., 2010), to compute total time spent in ‘recreational’ and ‘workplace’ physical activity (h/week).

### Analysis

Univariable linear regression was used to explore associations between AC and physical and mental wellbeing. We then adjusted for covariates in multivariable models. The final specification of these models was determined using Akaike's Information Criterion (AIC) to identify the models that best fit the data. Recognising the potential for weight status to act as a confounder or a mediator of the relationship between active commuting and wellbeing, we present models before and after its inclusion. All analyses were conducted in 2012 using R version 2.13.

## Results

Of the 1164 participants who completed the questionnaire, 128 were excluded from analysis due to physical disabilities or illnesses that may have prevented them from walking. A further 47 were excluded due to missing data in either outcome, exposure, or covariate measures. This resulted in a sample of 989 participants for analysis, of whom most were female (68%), educated to bachelor's degree level (73.1%) and neither overweight nor obese (65.1%) (Table 1). Median scores on SF-8 summary variables were higher than the population averages (50) for both physical (median = 56.0, IQR = 52.8–58.0) and mental (median = 52.5, IQR = 48.2–57.5) wellbeing.

AC, educational attainment, and recreational and workplace physical activity were all significantly associated with physical wellbeing in univariable and multivariable analyses (Table 2). There was a clear association between the amount of AC and physical wellbeing, but no such relationship was found for mental wellbeing (adjusted regression coefficients 0.29, 0.27 and 0.68 for 30–149 min/week, 150–224 min/week and ≥ 225 min/week respectively versus < 30 min/week, p = 0.52 for trend). After adjustment for covariates, the strength of the relationship between AC and physical wellbeing was attenuated slightly by the inclusion of weight status in the model. The final model (PCS model 2) suggested that higher physical wellbeing was associated with greater time spent in active commuting (adjusted regression coefficients 0.48, 0.79 and 1.21 for 30–149 min/week, 150–224 min/week and ≥ 225 min/week respectively versus < 30 min/week, p = 0.01 for trend). These findings differed very little in sensitivity analysis that omitted a small number of potentially influential cases (cases with standardised residuals < − 2 or > 2 for physical wellbeing (n = 46) and mental wellbeing (n = 60) models).

## Discussion

Our findings suggest that greater time spent actively commuting is associated with higher levels of physical wellbeing, independent of time spent in other domains of physical activity. In keeping with other studies of active commuting (Brown et al., 2004; Dunn et al., 2005), we found that the largest benefit was associated with participating in at least 45 min of active commuting per day. Although the adjusted regression coefficients of 0.48 and 1.21 points fall below the 3-point threshold for individual, ‘clinical’ significance in SF-8 summary measures (Bolge et al., 2009; Samsa et al., 1999), such differences may still have important population-level significance in settings such as Cambridge with a high prevalence of active commuting. However, contrary to studies of physical activity in general and to our own analysis of recreational physical activity, we found no evidence of a relationship between commuting and mental wellbeing (Hamer et al., 2009).

This study benefitted from the use of detailed physical activity data to explore the contribution of specific domains of physical activity (e.g. active commuting) to overall health and wellbeing, as encouraged by others (Morabia et al., 2012). However the cross-sectional design of this study is a key limitation: it is impossible to draw conclusions regarding the specific causal relationship between AC and physical wellbeing. It is also unclear how AC and weight status interact along the causal pathway, and what direction of causality (if any) underlies the strong association.

Finally, further studies are required to assess the generalisability of these findings. In particular, we have previously argued that almost all participants in this relatively affluent sample could potentially afford to travel by car or bus (Goodman et al., 2012). They could therefore determine their commuting practices in light of other non-financial considerations, including those of protecting their bodies from injury, over-exertion or the adverse effects of a sedentary lifestyle. It is possible that associations between AC and physical wellbeing would be less favourable in poorer settings where active travel may be imposed rather than chosen, and may be experienced as tiring or stressful (Bostock, 2001).

In conclusion, the findings presented here suggest that greater participation in active travel may contribute to improved health by increasing physical wellbeing. However, further longitudinal studies should examine the extent to which changing levels of active commuting and other forms of physical activity might contribute to improvements in overall health and wellbeing.

## Conflict of interest statement

The authors declare that there are no conflicts of interests.

## Figures and Tables

**Table 1:** Sample characteristics according to time spent in active commuting, Cambridge, UK (2009).

	Weekly time spent in active commuting			
	0–29 min	30–149 min	150–224 min	≥ 225 min
	n (%)	n (%)	n (%)	n (%)
*Gender (n = 989)*				
Male (n = 316)	64 (20.3)	101 (32.0)	84 (26.6)	67 (21.2)
Female (n = 673)	208 (30.9)	199 (29.6)	156 (23.2)	110 (16.3)

*Age (n = 989)*				
16–29 (n = 172)	36 (20.9)	53 (30.8)	56 (32.6)	27 (15.7)
30–39 (n = 291)	74 (25.4)	88 (30.2)	82 (28.2)	47 (16.2)
40–49 (n = 258)	76 (29.5)	81 (31.4)	52 (20.2)	49 (19.0)
50–59 (n = 201)	65 (32.3)	58 (28.9)	33 (16.4)	45 (22.4)
≥ 60 (n = 67)	21 (31.3)	20 (29.9)	17 (25.4)	9 (13.4)

*Highest educational qualification*				
Less than degree (n = 266)	99 (37.2)	80 (30.1)	52 (19.5)	35 (13.2)
Degree or higher (n = 723)	173 (23.9)	220 (30.4)	188 (26.0)	142 (19.6)

*Weight status*				
Normal or underweight (n = 644)	154 (23.9)	197 (30.6)	166 (25.8)	127 (19.7)
Overweight (n = 264)	78 (29.5)	86 (32.6)	58 (22.0)	42 (15.9)
Obese (n = 81)	40 (49.4)	17 (21.0)	16 (19.8)	8 (9.9)

*Physical component score (PCS-8)*				
Median (IQR)	55.3 (51.5–57.6)	56.0 (53.0–58.0)	56.2 (53.7–58.1)	56.3 (54.0–58.3)

*Mental component score (MCS-8)*				
Median (IQR)	52.3 (46.9–57.5)	52.5 (48.7–57.4)	52.5 (47.5–57.5)	52.7 (48.8–57.5)

*Recreational physical activity (h/week)*				
Median (IQR)	2.5 (0.0–5.0)	2.5 (0.0–5.0)	2.5 (0.0–5.0)	2.5 (0.0–5.0)

*Work-based physical activity (h/week)*				
Median (IQR)	5.1 (4.1–5.5)	5.3 (4.2–5.7)	5.3 (4.5–5.7)	5.3 (5.0–5.8)

*Distance from work (km)*				
Median (IQR)	20.9 (10.9–28.9)	4.8 (3.0–16.0)	4.8 (3.2–8.0)	8.0 (4.8–12.9)

Sample characteristics are presented as number of participants (n) with percentage of participants in parentheses (%). Descriptive statistics for PCS, MCS, physical activity and distance from work are presented as median and inter-quartile range (IQR) due to skewed distributions.

**Table 2:** Physical (PCS) and mental (MCS) wellbeing models, Cambridge, UK (2009).

Variables																			
		PCS unadjusted			PCS model 1^b^			PCS model 2^c^			MCS unadjusted			MCS model 1^d^			MCS model 2^e^		
		*B*	95% CI	p^a^	*B*	95% CI	p^a^	*B*	95% CI	p^a^	*B*	95% CI	p^a^	*B*	95% CI	p^a^	*B*	95% CI	p^a^
Gender	Male (reference)																		
	Female	− 0.3	(− 0.99, 0.40)	0.40	0.16	(− 0.56, 0.88)	0.40	0.13	(− 0.56, 0.88)	0.39	− **1**.**29**	(− **2**.**33**, −**0**.**25**)	**0**.**02**	− **0**.**96**	(− **2**.**00**, **0**.**09**)	**0**.**01**	− **1**.**04**	(− **2**.**09**, **0**.**01**)	**0**.**01**
Age (years)	16–29 (reference)																		
	30–39	− 0.71	(− 1.69, 0.27)	0.21	− 0.60	(− 1.57, 0.37)	0.20	− 0.43	(− 1.41, 0.54)	0.19	− 0.47	(− 1.92, 0.99)	< **0**.**01**	− 0.40	(− 1.86, 1.06)	< **0**.**01**	− 0.29	(− 1.76, 1.17)	< **0**.**01**
	40–49	− 0.59	(− 1.59, 0.41)		− 0.39	(− 1.40, 0.62)		− 0.18	(− 1.20, 0.83)		− 0.01	(− 1.50, 1.48)		− 0.04	(− 1.54, 1.47)		0.08	(− 1.44, 1.60)	
	50–59	− **1**.**13**	(− **2**.**20**, −**0**.**07**)		− 0.91	(− 1.97, 0.16)		− 0.60	(− 1.67, 0.48)		**2**.**16**	(**0**.**58**, **3**.**73**)		**2**.**16**	(**0**.**57**, **3**.**75**)		**2**.**32**	(**0**.**70**, **3**.**94**)	
	≥ 60	0.12	(− 1.34, 1.59)		0.39	(− 1.08, 1.86)		0.66	(− 0.82, 2.13)		**3**.**55**	(**1**.**37**, **5**.**73**)		**3**.**32**	(**1**.**13**, **5**.**51**)		**3**.**52**	(**1**.**31**, **5**.**73**)	
Active commuting	0–29 min/week (reference)																		
	30–149 min/week	0.78	(− 0.07, 1.62)	**0**.**01**	0.63	(− 0.22, 1.48)	**0**.**01**	0.48	(− 0.37, 1.33)	**0**.**01**	0.61	(− 0.67, 1.89)	0.49	0.26	(− 1.10, 1.73)	0.52	0.29	(− 1.07, 1.66)	0.52
	150–224 min/week	**1**.**19**	(**0**.**29**, **2**.**09**)		**0**.**92**	(**0**.**01**, **1**.**83**)		0.79	(− 0.12, 1.70)		0.57	(− 0.78, 1.93)		0.27	(− 1.20, 1.73)		0.27	(− 1.20, 1.73)	
	225 + min/week	**1**.**65**	(**0**.**67**, **2**.**63**)		**1**.**43**	(**0**.**44**, **2**.**42**)		**1**.**21**	(**0**.**22**, **2**.**21**)		1.15	(− 0.33, 2.63)		0.71	(− 0.83, 2.25)		0.68	(− 0.87, 2.23)	
Education	Less than bachelors degree (reference)													n.i			n.i		
	Bachelors degree or higher	**0**.**86**	(**0**.**13**, **1**.**58**)	**0**.**02**	0.68	(− 0.06, 1.43)	0.06	0.54	(− 0.20, 1.29)	0.05	− 0.05	(− 1.14, 1.05)	0.94						
Weight status	Normal/underweight (reference)				n.i.			− 0.52	(− 1.26, 0.23)	**0**.**01**	− 0.72	(− 1.83, 0.40)	0.45						
	Overweight	− 0.65	(− 1.38, 0.09)	< **0**.**01**	− **1**.**99**	(− **3**.**21**, −**0**.**77**)		− 0.16	(− 1.96, 1.65)		n.i.			− 0.97	(− 2.10, 0.16)	0.24
	Obese	− **2**.**51**	(− **3**.**69**, −**1**.**32**)								− 0.21	(− 2.04, 1.62)	
Total recreational physical activity	h/week	**0**.**11**	(**0**.**03**, **0**.**20**)	**0**.**01**	**0**.**13**	(**0**.**04**, **0**.**21**)	**0**.**01**	**0**.**11**	(**0**.**02**, **0**.**20**)	**0**.**01**	**0**.**20**	(**0**.**06**, **0**.**33**)	< **0**.**01**	**0**.**18**	(**0**.**05**, **0**.**31**)	**0**.**01**	**0**.**18**	(**0**.**04**, **0**.**31**)	**0**.**01**
Total work-based physical activity	h/week	**0**.**38**	(**0**.**14**, **0**.**63**)	< **0**.**01**	**0**.**34**	(**0**.**08**, **0**.**59**)	**0**.**01**	**0**.**35**	(**0**.**10**, **0**.**60**)	**0**.**01**	− 0.06	(− 0.42, 0.31)	0.77	n.i			n.i		
Distance from work	km	− 0.02	(− 0.05, 0.01)	0.15	n.i.			n.i.			− 0.04	(− 0.08, 0.00)	0.06	− 0.04	(− 0.09, 0.01)	0.13	− 0.03	(− 0.08, 0.02)	0.13

N.i. = variables not included in the model.

a p value for trend when the variable is entered continuously.

b Adjusted for gender, age, education, recreational PA and work-based PA.

c Adjusted for all variables in PCS model 1 plus weight status.

d Adjusted for gender, age, recreational PA, and distance from work.

e Adjusted for all variables in MCS model 1 plus weight status. Significant differences are shown in bold font.
